# The Influence of Antitumor Unsymmetrical Bisacridines on 3D Cancer Spheroids Growth and Viability

**DOI:** 10.3390/molecules26206262

**Published:** 2021-10-16

**Authors:** Jolanta Kulesza, Monika Pawłowska, Ewa Augustin

**Affiliations:** Department of Pharmaceutical Technology and Biochemistry, Chemical Faculty, Gdańsk University of Technology, 80-233 Gdańsk, Poland; jolanta.kulesza@pg.edu.pl (J.K.); monpawlo@pg.edu.pl (M.P.)

**Keywords:** spheroids, 2D and 3D cultures, cancer treatment, antitumor drugs, unsymmetrical bisacridines, cell death

## Abstract

The culture of 3D spheroids is a promising tool in drug development and testing. Recently, we synthesized a new group of compounds, unsymmetrical bisacridines (UAs), which exhibit high cytotoxicity against various human cell lines and antitumor potency against several xenografts. Here, we describe the ability of four UAs—C-2028, C-2041, C-2045, and C-2053—to influence the growth of HCT116 and H460 spheres and the viability of HCT116 cells in 3D culture compared with that in 2D standard monolayer culture. Spheroids were generated using ultra-low-attachment plates. The morphology and diameters of the obtained spheroids and those treated with UAs were observed and measured under the microscope. The viability of cells exposed to UAs at different concentrations and for different incubation times in 2D and 3D cultures was assessed using 7-AAD staining. All UAs managed to significantly inhibit the growth of HCT116 and H460 spheroids. C-2045 and C-2053 caused the death of the largest population of HCT116 spheroid cells. Although C-2041 seemed to be the most effective in the 2D monolayer experiments, in 3D conditions, it turned out to be the weakest compound. The 3D spheroid culture seems to be a suitable method to examine the efficiency of new antitumor compounds, such as unsymmetrical bisacridines.

## 1. Introduction

**Figure 1:**
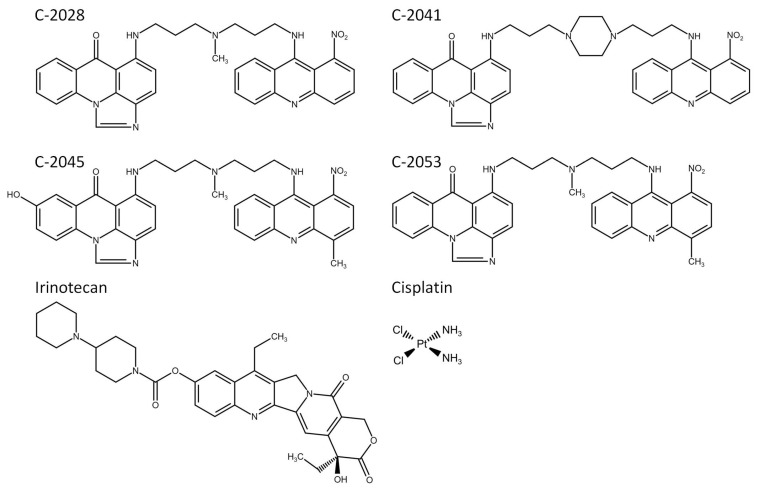
Chemical structures of the studied compounds: four unsymmetrical bisacridines (UAs), C-2028, C-2041, C-2045, and C-2053, and two reference compounds, irinotecan and cisplatin.

For many years, cancer has been the second leading cause of death in the world [1]. Therefore, a big challenge for modern medicine is the development of new, effective anticancer drugs, which, unfortunately, is a difficult task and involves a lot of costly and time-consuming research. To date, most in vitro studies with new chemotherapeutics have been carried out using traditional two-dimensional (2D) culture methods, in which cells grow in a monolayer. Cultures obtained this way are routinely used as initial models to evaluate the efficacy and safety of compounds with therapeutic potential [2,3,4]. Virtually all cells in the body exist in the three-dimensional (3D) environment, which is crucial for their growth, differentiation, and metabolism. Commonly used 2D systems cannot properly represent the function and phenotype of 3D tissues that are highly dependent on interactions with extracellular matrix proteins and neighboring cells [2,5]. 3D cell cultures (spheroids) serve as a link between the oversimplified 2D culture structure and the highly complex nature of tumors. Spheroids consist of different proliferation areas that are defined due to the nutrients, metabolites, pH, and oxygen gradients. These features, along with different gene expression patterns than in a monolayer culture, are similar to those found in poorly vascularized or avascular solid tumors [6,7,8]. This makes 3D spheroid cell culture systems useful models for the study of cancer biology, including anticancer drug testing. According to the majority of the literature reports, many compounds have clearly limited efficacy in 3D environments compared to the results obtained in 2D cultures. Therefore, spheroids are a good tool for the selection of chemotherapeutic agents with increased distribution and effectiveness in environments similar to in vivo conditions and may help to limit unnecessary animal testing [9,10]. However, generalizations about the drug resistance of cells in 3D versus 2D environments should not be made, as some potential molecular targets and signaling pathways play particular or even exclusive roles in the 3D environment and may be associated with increased activity of chemotherapeutic agents in spheroids compared with that in monolayer cultures [9]. Spheroids have been widely used for the in vitro biological evaluation of antitumor compounds; therefore, we decided to apply this culture model in our studies concerning unsymmetrical bisacridines (UAs) (Figure 1). This is a group of new, promising antitumor compounds among acridine derivatives, which has been patented in Europe and the USA [11,12], exhibiting high cytotoxic and antitumor activity against numerous cancers, including human lung and colorectal cancers [13]. They exhibited high cytotoxic activity against a set of 14 human tumor cell lines and antitumor activity against numerous xenografts in nude mice, including human lung and colorectal cancers, and also against Walker 256 adenocarcinoma in rats [13]. Two of the UA compounds, C-2028 and C-2045, were shown to induce apoptosis in HCT116 and H460 cells and accelerated senescence only in H460 cells [14].

In this study, we examined the effects of unsymmetrical bisacridines on the growth and morphology of tumor 3D spheroids derived from HCT116 and H460 cells. The influence of the studied bisacridines on cell viability in a 3D spheroid culture compared to that in a 2D culture system was also studied.

## 2. Results

### 2.1. Cytotoxicity of UAs and Reference Compounds in HCT116 and H460 Cells

Cytotoxic activity of unsymmetrical bisacridine derivatives against HCT116 and H460 cancer cell lines was evaluated using the MTT assay. For comparison, the cytotoxicity of irinotecan and cisplatin as reference compounds was also tested. Drug concentrations required to inhibit cell growth by 90% for UAs and by 50% for reference compounds are shown in Table 1. UAs inhibited the proliferation of both HCT116 and H460 cells at low concentrations, and the effects were similar for both cancer cell lines. C-2028 and C-2041 exhibited very high cytotoxic activity, with IC_90_ values not exceeding 0.046 and 0.049 µM, respectively, while C-2045 and C-2053 displayed lower cytotoxicity equal to around 0.4 and 0.2 µM, respectively. Both HCT116 and H460 cells were less sensitive to treatment with reference compounds than with UAs.

### 2.2. Morphology and Size of HCT116- and H460-Derived Spheroids

In the first step of our studies, we established proper seeding densities for generation of HCT116 and H460 spheroids. To achieve that, cell suspensions of HCT116 and H460 cells with various densities were seeded into ultra-low attachment (ULA) plates and their initial growth was monitored. The suggested range of diameters for conducting experiments with spheroids is 300–500 µm, preferably closer to 500 µm [15,16,17,18]; therefore, in further experiments for spheroid formation, a seeding density of 1 × 10^4^ cells/mL (2000 cells per well) for HCT116 and H460 cells was chosen (Figure S1 in the Supplementary Materials).

**Figure 2:**
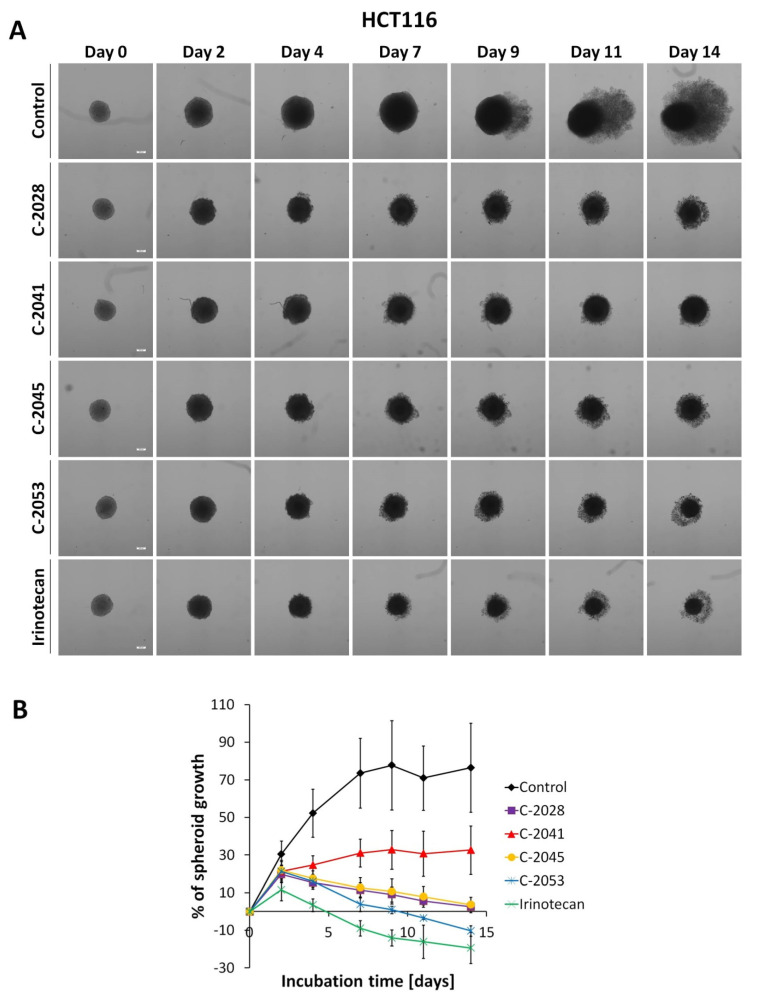
HCT116 spheroid morphology and kinetics. Spheroids were incubated with UAs at concentrations corresponding to IC_90_ values and irinotecan at an IC_50_ dose for 14 days. Every 2–3 days, images of the spheroids were taken and diameters were measured. (**A**) Representative images of the HCT116 control spheroids and spheroids treated with tested compounds. (**B**) Growth kinetics presented in a graph as percentages of spheroid growth over time. Data represent the averages of four independent experiments with standard deviation. White scale bars presented on images taken on day 0 correspond to 200 µm.

**Figure 3:**
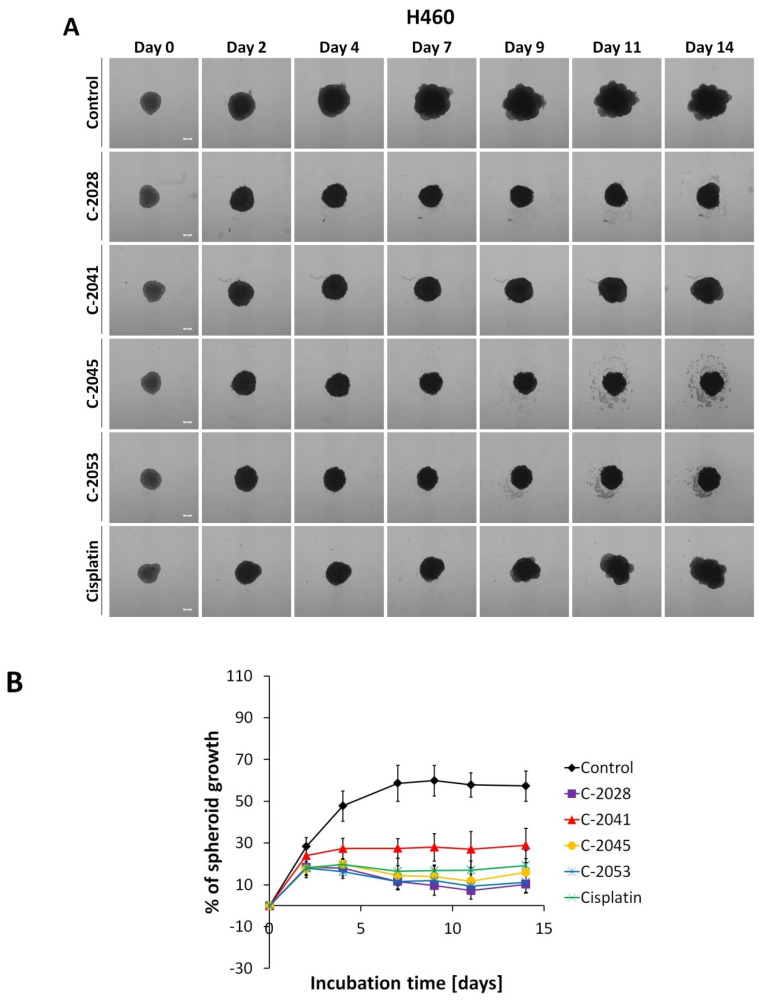
H460 spheroid morphology and kinetics. Spheroids were incubated with UAs at concentrations corresponding to IC_90_ values and cisplatin at an IC_50_ dose for 14 days. Every 2–3 days, images of the spheroids were taken and diameters were measured. (**A**) Representative images of the H460 control spheroids and spheroids treated with tested compounds. (**B**) Growth kinetics presented in a graph as percentages of spheroid growth over time. Data represent the averages of four independent experiments with standard deviation. White scale bars presented on images taken on day 0 correspond to 200 µm.

Microscopic observations of spheroids showed that both HCT116 and H460 cells formed spheroids with different morphometric features (Figure 2A and Figure 3A). The HCT116 spheres were more condensed, had a nearly perfect spherical shape, and a smooth, even periphery. H460 spheres in turn were bigger and presented a more irregular shape. Over time, control spheres of both cell lines showed slightly different growth kinetics. HCT116 spheres increased in size faster and reached bigger diameters. Spheroids derived from that cell line retained their circular rim up to day 7, and after 9 days of incubation, an incohesive peripheral cell layer appeared. In the case of H460 spheres, the growth was slower and to a lesser extent. Although lung spheroids kept a clearly defined periphery even after 14 days of incubation and no diffuse outer layer was observed, over time, H460 spheres evolved into heterogenous and lobular shapes.

Treatment of 3D spheroid cultures with the tested bisacridines and reference drugs caused visible changes in the morphology of the obtained spheroids. When incubated with the tested compounds, the smooth periphery of HCT116 spheres became jagged, and after prolonged exposure a diffuse outer layer of cells occurred. It was, however, more condensed than that of the cell layer present in control spheroids, and the core of HCT116 spheres treated with UAs and irinotecan was compact and clearly defined even after 14 days. In the case of H460 spheres after 9 days of incubation with cisplatin and 11 days with C-2041, spheroids began to take slightly lobular shapes similar to those observed in the control. Spheres incubated with C-2028, C-2045, and C-2053 did not show such features; they had condensed cores with cells sprouting at the periphery.

The most profound change after incubation with the tested compounds was the significant inhibition of their growth compared to that of untreated control cultures (Figure 2B and Figure 3B). In the case of HCT116 cells, after 4 days of incubation, all compounds except C-2041 showed a gradual reduction in spheroid size, with C-2053 exhibiting the greatest reduction. Spheroids incubated with this compound after 14 days were about 10% smaller than on day 0. A similar, slightly stronger effect was observed for the reference compound; the spheroids incubated with irinotecan after 14 days were almost 20% smaller in diameter than the spheroids on day 0.

On the other hand, H460 spheres began to decrease in size after 7 days of incubation with the UAs, and even after 2 weeks, neither compound caused a reduction of spheroids below baseline sizes. As observed in HCT116 spheres, in the case of H460-derived spheroids, treatment with C-2041 derivative inhibited the growth of spheroids to the smallest extent among the tested UAs. The remaining bisacridines, namely C-2028, C-2045, and C-2053, affected H460 spheres at similar levels. Moreover, spheroids incubated with those derivatives reached smaller sizes than they did with the reference compound, cisplatin.

### 2.3. Viability of Cells in 2D and 3D Cell Cultures of HCT116 and H460

**Figure 4:**
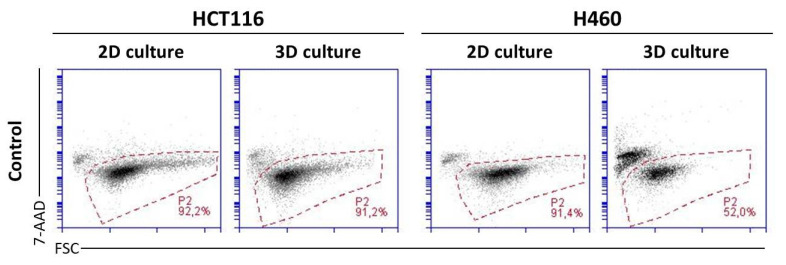
Viability of HCT116 (**left**) and H460 (**right**) cells cultured in 2D and 3D conditions. Cells in both culture systems were stained on day 3 with 7-AAD and subjected to flow cytometry analysis. P2: fraction of the 7-AAD negative cells (alive). Presented cytograms are representative of four independent experiments.

Flow cytometry analysis of HCT116 and H460 cells showed that both cell lines displayed high fractions of 7-AAD negative cells (alive) when grown in monolayers. Interestingly, HCT116 and H460 cell lines cultured in 3D conditions differed significantly in the number of viable cells (Figure 4). In the case of HCT116, both in monolayer culture and in spheroids, dead cells constituted less than 10% of the total population. In contrast, in H460-derived spheres, only about 52% of all cells were alive, which was a fraction almost 40% smaller than that in an adherent model of this cell line. In order to determine whether seeding density in the obtained spheroids has an impact on the percentage of alive cells in this culture model, further analysis was performed using spheroids derived from HCT116 and H460 cells seeded with different numbers of cells per well. In H460 spheres, regardless of seeding density, the number of alive cells was 52.3 ± 5.3%. Meanwhile, in HCT116 spheres, viable cells constituted 91.7 ± 1.9%.

The high percentage of dead cells (7-AAD^+^) in the H460 spheres makes the 3D model of this cell line not as suitable for analysis of the cellular response induced by anticancer compounds as the 3D model of the HCT116 cell line. Therefore, the H460 spheroid model was not used in further experiments regarding treatment with UAs.

**Figure 5:**
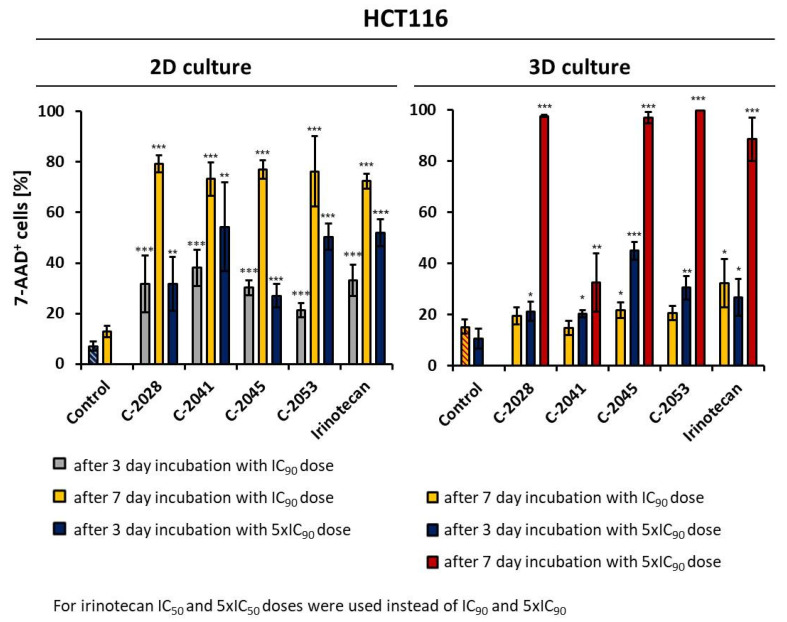
Effects of C-2028, C-2041, C-2045, C-2053, and irinotecan on cell viability in HCT116 cells cultured in 2D and 3D conditions. Cells were incubated with tested compounds at concentrations corresponding to IC_90_ and 5× IC_90_ values (IC_50_ and 5× IC_50_ for irinotecan) for 3 or 7 days, stained with 7-AAD, and subjected to flow cytometry analysis. Bar graphs show quantified data, expressed as the percentages of 7-AAD^+^ (dead) cells after incubation with the tested compounds in 2D (**left**) and 3D (**right**) cell cultures. Data are presented as the means ± SD of at least three independent experiments. Significant differences in cell percentages between the control and cells treated with the compound are indicated as follows: * *p* < 0.05; ** *p* < 0.01; *** *p* < 0.001.

HCT116 cells cultured in a monolayer and as spheroids were incubated for 3 or 7 days with tested compounds at concentrations corresponding to the IC_90_ or 5× IC_90_ values for UAs and IC_50_ or 5× IC_50_ values for irinotecan. Then, cells were stained with 7-AAD and analyzed by flow cytometry. The obtained results showed that 2D and 3D culture models differed in the intensity of the observed cellular response (Figure 5).

When grown in a monolayer culture, HCT116 cells, after 3 days of treatment with UAs at IC_90_ doses, showed at least three-fold higher percentages of dead cells than did the untreated control. Among the tested compounds, the C-2041 derivative induced cell death in HCT116 cells to the highest extent; after 72 h of incubation with a concentration corresponding to an IC_90_ value, the fraction of non-viable cells reached 38.1%. C-2028 and C-2045 were less potent and affected colorectal cancer cells on a similar level to irinotecan, with the proportion of 7-AAD^+^ cells being just above 30%. In the case of the C-2053 derivative, after 72 h of incubation with this compound, only 21.4% of all the HCT116 cells were dead.

Since 3 days of incubation with UAs and irinotecan at IC_90_ and IC_50_ doses had a moderate effect on HCT116 cells grown in a monolayer, we decided not to analyze the cellular response under these conditions in spheroids. Instead, we selected two alterations for further studies: we extended the incubation time to 7 days and increased the doses of the tested compounds five-fold.

For both UAs and irinotecan, the fractions of 7-AAD^+^ cells (dead) were much higher in 2D culture than they were in 3D culture after 7 days of incubation with the compounds at IC_90_ doses. In a monolayer culture, over 70% of cells incubated with the tested compounds were non-viable, while in spheroids, these fractions reached only 19.5, 14.8, 21.8, 20.5, and 32.3% for C-2028, C-2041, C-2045, C-2053, and irinotecan, respectively. Subsequently, the treatment with compounds at a 5× IC_90_ dose resulted in a similar relationship: after 3 days of incubation, the amount of dead cells was higher in the 2D culture than in the 3D culture. The biggest difference was visible in the case of the C-2041 derivative, where in 2D culture after 72 h of exposure, the fraction of dead cells was 52%, which was over 2.5 times higher than that for the 3D culture (20.3%). The only exception was the treatment of the cells with C-2045, where in 2D culture, 27% of all the cells were dead, while in spheroids, this population reached almost 45%, suggesting that this compound can be more effective in 3D culture.

Extending the incubation time from 3 to 7 days at 5× IC_90_ for 3D cultures resulted in a marked increase in the number of 7-AAD^+^ cells. These conditions were not applied to cells grown in a monolayer due to the fact that prolonged incubation time with high doses of compounds is difficult to maintain in monolayer cultures. In adherent models, cells are evenly exposed to the equal concentration of tested compound, in contrast to spheroids and in vivo tumors, where a characteristic gradient is present. Thus, prolonged treatment with high doses of compounds in 2D conditions would result in the massive death of cells, which would cause problems when collecting them for further analysis. In HCT116 spheres, after 7 days of incubation with 3 tested bisacridines (C-2028, C-2045, and C-2053 at 5× IC_90_ doses) the fraction of non-viable cells was very high, reaching over 96%. A slightly weaker effect was observed for irinotecan, where after incubation with a 5× IC_50_ dose for 7 days, 88.4% of cells were dead. Surprisingly, for HCT116 spheroids undergoing treatment with the C-2041 derivative, even after 7 days of exposure, more than 67.5% of all the cells remained viable. In these conditions, only 32.5% cells were dead, which was still less than for the non-viable population of cells treated with an IC_90_ dose of C-2041 for 3 days in a 2D monolayer culture (38%).

## 3. Discussion

One of the principal goals in pharmacy and medicine for many years has been the development of effective and harmless anticancer drugs. Another aim has been to construct more predictable cellular models for testing drug sensitivity and to limit the usage of animals in the evaluation of the pharmacokinetic properties of studied compounds. Classic methods based on 2D monolayer cell cultures are very useful tools to show the biochemical and molecular effects of a new compound, but these cannot show the possibility and efficiency of a drug in penetrating the tumor and its possible action in the patients’ tissues and body. Three-dimensional (3D) cultures are becoming significant additions in testing and detecting valuable drug candidates and are turning into essential tools in anticancer drug research [19].

Here, we present a comparison of action for four new unsymmetrical bisacridines derivatives in (i) monolayer culture (2D) and (ii) spheroids, resembling the 3D environment. We checked whether UA compounds can affect spheroids of HCT116 and H460 cells and inhibit their growth, and whether they can induce cell death in standard monolayer culture and in spheroids. Both studied human cancer cell lines, colorectal HCT116 and lung H460, were capable of creating spheroids. According to the 2017 classification of 60 human cancer cell lines by Selby et al., spheroids were divided into four categories based on their morphology and the degree of intercellular adhesion. Spheroids formed by HCT116 cells were classified into the first group, i.e., condensed spheroids—tight and round spheres with smooth and even edges. In turn, the H460 spheres were classified into the second group, i.e., non-condensed spheroids—generally rounded spheroids with a rougher perimeter [20]. The morphology of the spheroids obtained in our laboratory is consistent with this classification and observation.

The evaluation of the survivability of cells in the control spheroids revealed that H460 spheres consisted of very high numbers of dead cells: Only 52% of cells 3 days after the generation of spheroids remained alive, while at the same time HCT116 spheres contained more that 90% alive cells. It is well known that the cores of spheroids, due to weaker diffusion of nutrients and the gradation of oxygen, may consist of many dead cells [21]. However, further maintenance of spheroid culture and their additional exposure to cytotoxic compounds would cause more intensive cell death; it is hard to distinguish the mechanism of action of the drug from the natural behavior of the spheroids. Furthermore, preliminary experiments on UA-treated H460 spheroids stained with 7-AAD uncovered the difficulty in conducting cytometric analysis. With respect to the above, we did not perform broad experiments with H460 spheres but focused mainly on studies with HCT116 cells.

An important feature of all tested UA derivatives (C-2028, C-2041, C-2045, and C-2053) is that they exhibited very high cytotoxicity in monolayer culture against HCT116 and H460 cancer cells, with similar levels for both cell lines (IC_90_ values ranged from 0.04 to 0.4 µM) (Table 1). C-2028 and C-2041 compounds were slightly more active than C-2045 and C-2053. All compounds managed to inhibit cell proliferation at very low concentrations, much lower than that of the reference compounds, for which IC_50_ values amounted to 4.5 and 2.5 µM for irinotecan against HCT116 cells and cisplatin against H460 cells, respectively. The obtained cytotoxicity results for the reference drugs are comparable to those found by other laboratories [22,23,24]. UA compounds were also more effective than other drugs that are frequently used in clinics, such as oxaliplatin applied in colon tumor treatment [25] or etoposide and paclitaxel used in lung cancer chemotherapy [26,27].

Studies with HCT116 spheres showed that the four UA derivatives behaved differently in 3D cultures than in 2D cultures. The best way to evaluate a drug’s influence on spheroids is to measure the diameters of the spheroids exposed to the compound with increasing time of treatment. Three compounds, namely C-2028, C-2045, and C-2053, distinctly managed to stop the growth of HCT116 spheres, to a slightly smaller degree than did the reference compound, irinotecan (Figure 2A). Importantly, colon spheroids treated with C-2053 after 10 days became even smaller than the initial untreated spheroids, suggesting that this compound can affect and inhibit the proliferation of cells in 3D cultures to the highest extent among UA derivatives. Cytotoxicity carried out on a 2D monolayer culture of HCT116 cells did not indicate C-2053 as the compound with the strongest influence on the spheroids’ diameter. Experiments conducted with H460 spheres also showed that C-2028, C-2045, and C-2053 derivatives were very effective against spheroids and inhibited their growth even more than the reference compound, cisplatin, did. However, none of these compounds managed to reduce the size of H460 spheroids below the diameter of the initial untreated spheroids, in contrast to what occurred in HCT116 cells. This is not the first time that cisplatin exhibited poor effects on a spherical culture [23].

Furthermore, the C-2041 derivative caused the smallest decrease in spheroid diameter of both cell lines, HCT116 and H460. Surprisingly, experiments carried out on the 2D monolayer culture suggested that C-2041 should be the compound with the strongest impact on cell viability (Figure 5, left panel). 7-AAD staining of cells cultured as spheroids revealed that only 32.5% of HCT116 cells were dead after 7 days of exposure to a 5 × IC_90_ concentration of C-2041. At the same time, other compounds caused the death of at least 90% of cells.

Interestingly, C-2045 did not seem to be the compound that caused cell death of the largest number of cells in the HCT116 sphere culture (Figure 5). C-2045 at a 5× IC_90_ dose after 3 days of exposure caused cell death in sphere culture, even in a bigger population of cells than in the monolayer. This suggests that C-2045 can be a very effective compound for in vivo studies. Therefore, performing parallel studies in 2D monolayer culture with 3D spheroids would better reflect the effectiveness and validity in undertaking further research on our new group of drugs. The most effective bisacridine derivative in the 3D spheroid culture was C-2045, followed by C-2053 and C-2028, while C-2041 turned out to have a very weak influence on spheroid size and viability.

It is worth mentioning that our bisacridine derivatives were tested against human xenografts on nude mice. The antitumor potency of C-2028 and C-2045 was evaluated in HCT116 cells, whereby both compounds managed to significantly inhibit growth of the tumor [14]. C-2028 was more potent in this research than was the C-2045 derivative; however, the same dosage of drugs was used in the mice treatment, although C-2045 exhibited lower cytotoxicity and underwent glucuronidation, which might have decreased the effective drug concentrations in mice [28]. The therapeutic effectiveness of the four bisacridine derivatives was also evaluated in the panel of several human tumor xenografts in nude mice [13]. All the compounds, including C-2041, displayed high tumor growth inhibition index (TGI) values, especially against pancreatic cancer cells, such as PANC-1, Mia-Pa-Ca-2, BxPC-3, and other cell lines, which are characterized by slow growth rates. C-2053 had to be administrated in high doses to achieve the same effect as the other derivatives, confirming our observations presented in this research. Only the antitumor potency of C-2028 was assessed in HCT116 and H460 xenografts (fast tumor growth), and the compound inhibited the growth of tumors by around 30%, which was much weaker than in experiments with pancreatic cells. It is worth mentioning that, although C-2041 managed to stop the growth of xenografts to a similar extent to the other tested derivatives, it displayed very low maximal tolerated doses, suggesting high toxicity for this compound, which may result in difficulties in the usage of this compound in clinics [13].

An increasing number of studies have presented results obtained from comparisons of the effectiveness of drugs in 2D and 3D cell culture models. They usually refer to newly synthesized compounds, such as taxoid SB-T-1214 [29], ruthenium complex KP1339/IT-139 [30], or tryptophan-rich peptide P1 [31], or natural extracts that may exhibit anticancer properties, such as Oliviera essential oils [32] or curcumin [33]. Researchers often apply 3D cultures to test new combinations of clinically used drugs or conjugates of these drugs with nanoparticles [34]. For example, HT29 spheroids were used to determine drug responses and highlight the effects of the drugs oxaliplatin, 5-fluorouracil, and folinic acid following the encapsulation of their respective liposomes [35]. In other studies, tumor spheroids derived from ovarian cancer cell lines or primary patients’ cells were sensitized to cisplatin treatment by using an inhibitor of A disintegrin and metalloprotease 17 (ADAM17), named GW280264X, to overcome resistance [36]. In summary, the application of 3D models has the potential to improve drug discovery research and bridge the gap between results obtained in preclinical phases and promising outcomes found in clinical trials.

In conclusion, three UA derivatives, C-2028, C-2045, and C-2053, were very effective against cancer cells, both when grown in monolayer cultures and when forming spheroids. In contrast, C-2041 did not have as strong of an impact on the cellular response in 3D spheroid culture as it did in 2D culture. Applying 3D models for the study of the cellular effects triggered by our novel group of unsymmetrical bisacridine derivatives demonstrated their attractive and significant features.

## 4. Materials and Methods

### 4.1. Tested Compounds

The unsymmetrical bisacridines (UAs) C-2028, C-2041, C-2045, and C-2053 were synthesized as methanosulphonians (C-2028, C-2041, and C-2045) or monochloride (C-2053) in the Department of Pharmaceutical Technology and Biochemistry, Gdańsk University of Technology, according to a previously published procedure [13]. Both stock and working solutions were prepared in sterile deionized Mili-Q water (Merck/Sigma-Aldrich, Darmstadt, Germany). The reference compounds irinotecan and cisplatin were purchased from Sigma-Aldrich (St. Louis, MO, USA) and the stock solution was prepared in dimethyl sulfoxide (DMSO; POCH S.A, Gliwice, Poland), while working solutions were prepared in sterile, deionized Mili-Q water.

### 4.2. Cell Lines and Culture Conditions

Human colorectal carcinoma HTC116 and non-small-cell lung carcinoma H460 cells were purchased from the American Type Culture Collection (ATCC, Manassas, VA, USA) and were tested negatively for mycoplasma using the Universal Mycoplasma Detection Kit (ATCC, Manassas, VA, USA). HCT116 cells were cultured in McCoy’s 5A medium (Sigma-Aldrich, St. Louis, MO, USA) and H460 cells in RPMI 1640 medium (Sigma-Aldrich, St. Louis, MO, USA). Both media were supplemented with 10% heat-inactivated fetal bovine serum (FBS; Biowest, Nuaille, France), 100 µg/mL streptomycin, and 100 U/mL penicillin. Cells were incubated at 37 °C in 5% CO_2_ atmosphere. All the experiments were carried out with cells in the exponential phase of growth.

### 4.3. Cell Growth Inhibition Assay

To estimate cell viability, the MTT assay was used. HCT116 and H460 cells were seeded in 24-well plates with 2 × 10^4^ cells per well. After 24 h of incubation at 37 °C in 5% CO_2_ atmosphere, unsymmetrical bisacridines or reference compounds (irinotecan/cisplatin) were added at concentrations up to 10 µM for UAs and up to 200 µM for reference compounds. After 72 h of incubation, 200 µL/well of 3-(4,5-dimethylthiazol-2-yl)-2,5-diphenyltetrazolium bromide (MTT; Abcam, Cambridge, Great Britain) at a concentration of 4 mg/mL was added and incubated for 3 h at 37 °C. Next, the culture medium from each well was removed, the formazan crystals were dissolved in DMSO, and absorbance at 540 nm was measured. The concentrations of drugs required for inhibition of cell growth by 90% (IC_90_) for UAs and 50% (IC_50_) for reference compounds compared with untreated control cells were calculated from the curves plotting survival as a function of dose. Results were obtained from at least four independent experiments (n = 4).

### 4.4. Establishment of Seeding Density for Spheroid Formation

For spheroid formation, 96-well Corning^®^ Costar^®^ Ultra-Low Attachment (ULA) round-bottomed plates (Corning Incorporated, Kennenbuck, ME, USA) were used. After trypsinization and counting, cell suspension was centrifuged in order to remove trypsin and fresh culture medium was added to dissociate the pellet. Then, 200 µL/well of HCT116 or H460 cell suspensions at various densities (0.5 × 10^4^, 1 × 10^4^, 1.25 × 10^4^, 1.5 × 10^4^, 2 × 10^4^, 2.5 × 10^4^ cells/mL (1000, 2000, 2500, 3000, 4000, 5000 cells/well)) were dispensed into the ULA plate using a multichannel pipette. After seeding, the plate was centrifuged for 15 min at 1200 rpm at room temperature to initiate cell aggregation and then incubated at 37 °C in a 5% CO_2_ atmosphere for 3 days to enable formation of spheroids. Afterwards images of generated spheroids were taken using a 4× objective in an OLYMPUS IX 83 inverted microscope with XC 50 camera and cellSens Dimension software. Then, 100 µL of medium in each well was carefully replaced with fresh medium and this day was further referred to as day 0. Images of spheroids were taken daily for the next three days; the diameter of each spheroid was measured and the mean value was calculated.

### 4.5. Generation of Tumor Spheroids

Cells were trypsinized, counted, centrifuged, and suspended in fresh culture medium. Then, 200 µL/well of HCT116 or H460 cell suspension at a density 1 × 10^4^ cells/mL (2000 cells per well) was dispensed into the ULA plate and the plate was centrifuged for 15 min at 1200 rpm at room temperature. Afterwards, the plate was incubated at 37 °C in a 5% CO_2_ atmosphere for 3 days before further experiments.

### 4.6. Spheroid Size and Morphology Assessment

The spheroids were generated as described above. First, 72 h after seeding, images of each spheroid were captured using a 4× objective in an OLYMPUS IX 83 inverted microscope with XC 50 camera and cellSens Dimension software. Afterwards, 100 µL of culture medium was carefully replaced with fresh medium in control spheroids, or fresh medium with 0.04, 0.05, 0.4, 0.2, 4.5, and 3.0 µM of C-2028, C-2041, C-2045, C-2053, irinotecan, and cisplatin, respectively. Images of spheroids were taken every 2–3 days up to 14 days after drug treatment, and each time the diameters of spheroids were measured using the cellSens Dimension software. Results were obtained from four independent experiments (n = 4). For each compound, at least 8 spheroids were measured during each experiment and the mean value of the spheroid growth was calculated as shown below:

$$\%\;\mathrm{spheroid}\;\mathrm{growth}=\frac{d_{x}}{d_{0}}*100\%$$

where d_x_ is the mean diameter of at least 8 spheres at a given day of incubation and d_0_ is the mean diameter of at least 8 spheres at day 0 (day of the drug treatment).

### 4.7. Cell Death Assay

For cell death evaluation, 7-aminoactinomycin D (7-AAD) dye (Thermo Scientific, Waltham, MA, USA) was used. In monolayer cultures, 1 × 10^6^ cells were seeded on a 100 mm plate (1 × 10^5^ cells for 3 days of and 1 × 10^4^ for 7 days of untreated control) and allowed to adhere overnight. Then, cells were treated with UAs at concentrations corresponding to IC_90_ values (0.04, 0.05, 0.4, and 0.2 µM for C-2028, C-2041, C-2045, and C-2053, respectively) or 5× IC_90_ values, while reference compounds were added at concentrations corresponding to IC_50_ (4.5 and 3.0 µM for irinotecan and cisplatin) or 5× IC_50_ values for 3 or 7 days. After drug treatment and trypsinization, 0.5 × 10^6^ cells were collected from plates, centrifuged at 1000 rpm for 5 min at RT, washed twice with PBS, pelleted and resuspended in 150 µL of PBS, and stained with 7-AAD dye (1 µg/mL) for 15 min in the dark at RT. Spheroids were generated as described above; then, 72 h after seeding, 100 µL of culture medium was changed and cells were treated for 3 or 7 days with drugs at concentrations corresponding to IC_90_ or 5× IC_90_ values for UAs and IC_50_ or 5× IC_50_ values for reference compounds. After drug treatment, spheroids were disaggregated to obtain a single-cell suspension for flow cytometry analysis. To accomplish this, spheroids were collected, centrifuged, washed with PBS, and treated with 200 µL of trypsin and pipetted to promote cellular detachment. Next, fresh medium was added to neutralize trypsin, cells were centrifuged, washed twice with PBS, suspended in 150 µL of PBS, and stained with 1 µg/mL 7-AAD for 15 min in the dark at RT. After staining, the cells were analyzed using flow cytometry with FACS Accuri C6 (BD, San Jose, CA, USA) and the data were analyzed using the BD AccuriTM C6 Software Version 1.0.264.21. Each experiment was repeated at least three times (n = 3). Each time at least 10,000 cells were subjected to flow cytometry analysis. The acquisition gates were restricted to cell gates based on morphological characteristics (FSC vs SSC). 7-AAD was excited at 488 nm and its fluorescence was analyzed at 585/40.

### 4.8. Statistical Analysis

The results are presented as means ± SD of at least three independent experiments. Statistical analysis was performed by the Student’s *t*-test, and the differences of *p* < 0.05 between the two groups were considered statistically significant: * *p* < 0.05, ** *p* < 0.01, *** *p* < 0.001.

## 5. Conclusions

In this study we focused on the influence of four newly synthesized antitumor compounds, unsymmetrical bisacridines (UAs), on the growth and viability of spheroids derived from colon HCT116 and lung H460 cells. All derivatives, C-2028, C-2041 C-2045, and C-2053, exhibited high cytotoxicity against both studied cell lines grown in a monolayer. HCT116 and H460 cells were able to form spheroids, whose size and the growth rate depended on the seeding density. UAs treatment inhibited the growth of HCT116 and H460 spheroids; three compounds, C-2028, C-2045, and C-2053, greatly reduced the size of the spheroids, similarly to the reference drugs irinotecan and cisplatin, while C-2041 was less potent in spheres-growth inhibition. The viability of cells in spheroids was tested in HCT116 spheres and again C-2028, C-2053, and especially C-2045 were very effective in cell death induction, whereas C-2041 was much weaker.

In conclusion, UA compounds, particularly C-2045, C-2053, and C-2028, are very potent against three-dimensional cultures of tested cell lines and should be the subject of extended studies. C-2041 due to its limited properties in affecting spheroids growth and viability may not be efficient in experiments on animals or in clinical trials.

## Figures and Tables

**Table 1:** IC_90_ values for UAs (C-2028, C-2041, C-2045, and C-2053) and IC_50_ values for reference compounds (irinotecan and cisplatin).

Compound	Drug Dose	Drug Concentration [µM]	
		HCT116	H460
C-2028	IC_90_	0.044 ± 0.005	0.046 ± 0.004
C-2041	IC_90_	0.049 ± 0.005	0.046 ± 0.005
C-2045	IC_90_	0.455 ± 0.026	0.399 ± 0.052
C-2053	IC_90_	0.195 ± 0.075	0.184 ± 0.022
Irinotecan	IC_50_	4.515 ± 0.304	Not applicable
Cisplatin	IC_50_	Not applicable	2.535 ± 0.627

## Data Availability

The data presented in this study are available on request from the corresponding author.

## Supplementary Materials

The following are available online. Figure S1: Establishment of seeding conditions for HCT116 and H460 spheroid formation. Cell suspensions with different densities were seeded into ULA plates and incubated for 72 h to allow spheroid formation. Then for 4 subsequent days images of spheroids were taken and diameters measured. (A) Representative microscopic imaged of HCT116 (left) and H460 (right) spheroids obtained from various seeding densities. (B) HCT116 (left) and H460 (right) initial tumor spheroid growth curves. Values are mean ± SD.

## Sample Availability

Samples of the compounds C-2028, C-2041, C-2045, and C-2053 are available from the authors.
