# Supplementary material for: The Influence of Antitumor Unsymmetrical Bisacridines on 3D Cancer Spheroids Growth and Viability
Source: Molecules. 2021 Oct 16;26(20):6262. doi: 10.3390/molecules26206262 (PMC8538688; doi:10.3390/molecules26206262)
Supplement: Supplementary file 1 [file molecules-26-06262-s001.zip › molecules-1356187-supplementary.pdf]

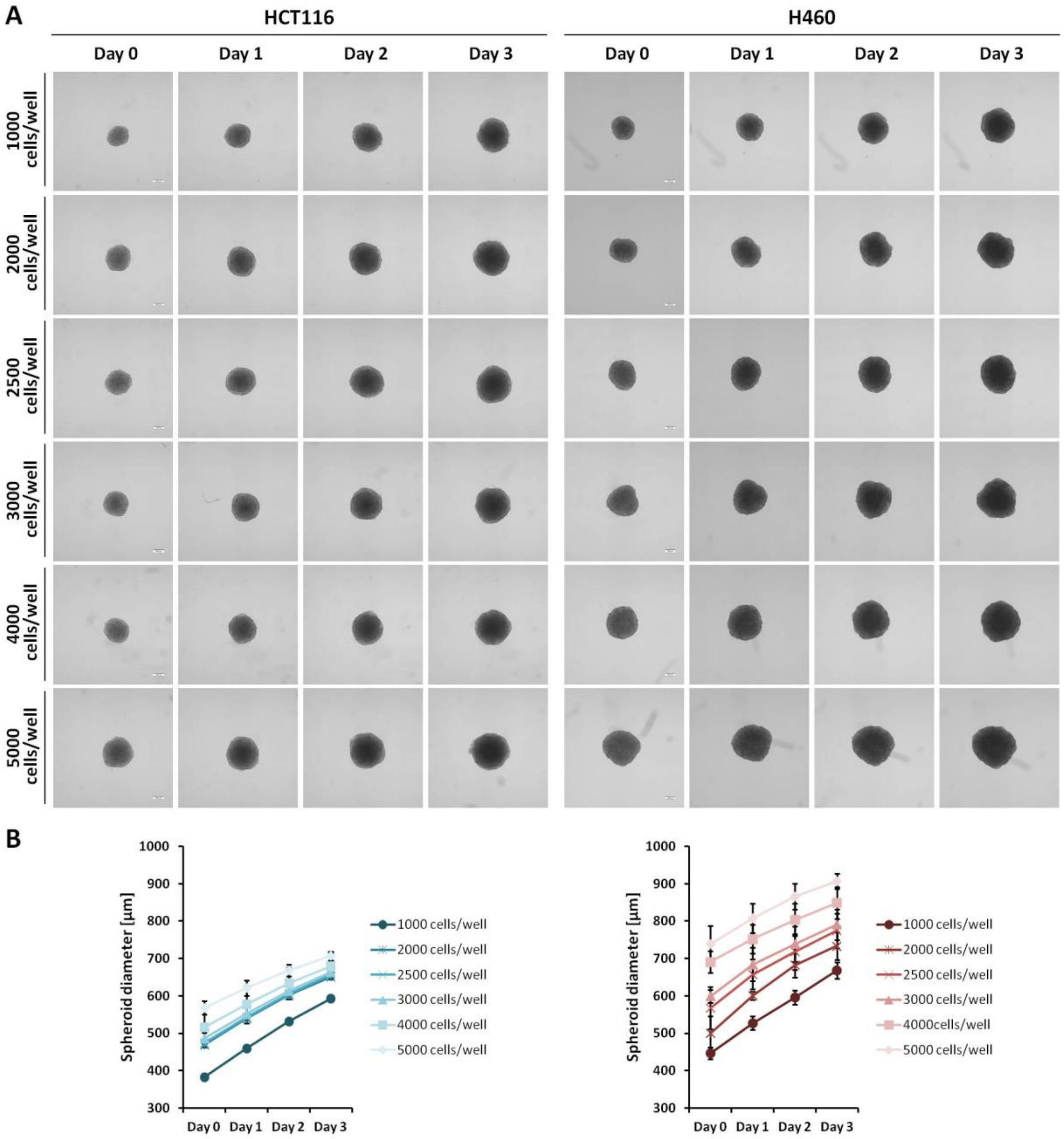

Figure S1. Establishment of seeding conditions for HCT116 and H460 spheroid formation. Cell suspensions with different densities were seeded into ULA plates and incubated for 72h to allow spheroid formation. Then for 4 subsequent days images of spheroids were taken and diameters measured. **(A)** Representative microscopic imaged of HCT116 (left) and H460 (right) spheroids obtained from various seeding densities. **(B)** HCT116 (left) and H460 (right) initial tumor spheroid growth curves. Values are mean ± SD.
